# Hierarchical modeling in association studies of multiple phenotypes

**DOI:** 10.1186/1471-2156-6-S1-S104

**Published:** 2005-12-30

**Authors:** Xin Liu, Eric Jorgenson, John S Witte

**Affiliations:** 1 Department of Epidemiology and Biostatistics, University of California, San Francisco, CA 94143-0560, USA

## Abstract

The genetic study of disease-associated phenotypes has become common because such phenotypes are often easier to measure and in many cases are under greater genetic control than the complex disease itself. Some disease-associated phenotypes are rare, however, making it difficult to evaluate their effects due to small informative sample sizes. In addition, analyzing numerous phenotypes introduces the issue of multiple comparisons. To address these issues, we have developed a hierarchical model (HM) for multiple phenotypes that provides more accurate effect estimates with a lower false-positive rate. We evaluated the validity and power of HM in association studies of multiple phenotypes using randomly selected cases and controls from the simulated data set in the Genetic Analysis Workshop 14. In particular, we first analyzed the association between each of the 12 subclinical phenotypes and single-nucleotide polymorphisms within the known causal loci using a conventional logistic regression model (LRM). Then we added a second-stage model by regressing all of the logistic coefficients of the phenotypes obtained from LRM on a **Z **matrix that incorporates the clinical correlation of the phenotypes. Specially, the 12 phenotypes were grouped into 3 clusters: 1) communally shared emotions; 2) behavioral related; and 3) anxiety related. A semi-Bayes HM effect estimate for each phenotype was calculated and compared with those from LRM. We observed that using HM to evaluate the association between SNPs and multiple related phenotypes slightly increased power for detecting the true associations and also led to fewer false-positive results.

## Background

Complex diseases are most commonly evaluated in association studies as a single phenotype. However, they often comprise numerous phenotypes with varying degrees of interrelatedness. For example, in the study of alcoholism, brain electrophysiological measures (e.g., electroencephalograms and event-related potenials) can be evaluated as biological markers for developing alcoholism. Focusing on such disease-associated phenotypes can help improve a study if they are under greater genetic control or easier to measure than the ultimate disease endpoint (e.g., alcoholism). Nevertheless, analyzing the effect of genes on numerous phenotypes introduces issues of multiple comparisons, and can lead to imprecise estimates of association if the number of individuals exhibiting a disease-associated phenotype is limited.

These issues can be addressed by using a hierarchical model (HM) that compromises between analyses of a single phenotype and numerous disease-associated phenotypes. Specifically, this approach shrinks conventional estimates for phenotypes toward a prior mean distinguished by their biologically interrelated nature. Previous work has shown that HM can improve conventional estimation of the association between disease(s) and exposures [1-4], fine-mapping by linkage disequilibrium modeling [5], and gene × environment interaction [6-8]. Here, we show how HM can be used to evaluate the association between genetic factors and multiple disease-associated phenotypes, and evaluate the performance of HM by comparing power and false-positive rates (FPR) with a conventional logistic regression model (LRM).

## Methods

We used the simulated dataset from the Genetic Analysis Workshop 14 (GAW14) to sample cases and controls for association analyses. Specially, we randomly selected 1 case per family across 4 populations (Aipotu, Karangar, Danacaa, New York City), which gives a total of 350 cases per replicate. Then 50 controls per replicate were pooled together and used as a "population" (*n *= 5,000) for control selection. In light of the limited number of cases, we selected two controls per case, leading to a total of 1,050 subjects per replicate (350 cases, 700 controls).

For the 12 phenotypes, we first analyzed each using a conventional logistic regression model:

logit (Pr (*Y*_m _= 1) | *g*^T^) = μ_m _+ *g*^T^β_m _+ sex β_sex_,     (1)

where binary traits *Y*_1_... *Y*_m _(m = 12) refer to the phenotypes *a *- *l *in the simulated data, and *g *is the single-nucleotide polymorphism (SNP)-specific coding for each individual. For simplicity's sake, we assumed a dominant model for the minor allele, but not for D6, because its common allele is truly functional. Sex was included in Equation 1 because of its potential role in psychiatric disorders. Our HM attempted to improve the first-stage estimates of the SNP-effects on the phenotypes (β_m_) by adding the following second-stage linear regression model:

β = **Z**π + *U*     (2)

where *U* ~ *N*_m _(**0**_m_, τ ^2^). Here, **Z **is a 12 × 3 matrix containing second-stage covariates defining 3 clusters of the 12 phenotypes: 1) communally shared emotions; 2) behavioral related; and 3) anxiety related. In particular, for each phenotype, the corresponding elements in **Z **are set equal to 1 if they have been defined as being within one of the clusters, and 0 otherwise. π is a column vector of coefficients corresponding to the effects of the second-stage covariates on the SNP (i.e., cluster-level effects). In this way, **Z **incorporates into a second stage the clinical correlation of the phenotypes, whereby the estimate of each β_m _"borrows" information from the other estimates. We fixed τ as 0.354, using a semi-Bayes HM (in empirical-Bayes one would estimate the τ). This value implies a four-fold range of residual odds ratios for the SNP on phenotypes (exp(± 3.92*0.354)) after accounting for the relations defined in **Z **[5]. Finally, we compared the estimates of phenotype effects obtained from the conventional LRM approach (Equation 1) to those obtained from HM (Equations 1 and 2 combined).

To compare the models, we bought 10 packets of markers, which contained 4 disease loci and 2 modifier genes. Four markers were chosen as the causal surrogates because they either provided the highest power compared with other SNPs in the same region (D1, D3, and D4) or had higher power while also being informative for the power comparison (i.e., with less than 100% power for both the HM and LRM (D2)). Together with two modifier genes, which were defined as D5 and D6, a total of 6 SNPs were used for the power comparison. We calculated power as the proportion of replicates in which the tested SNP was statistically significantly associated with a phenotype at an alpha level = 0.05.

A total of 190 SNPs were used for the type I error comparisons (excluding 10 microsatellite markers). Phenotype *i *and *j *were simulated randomly, so any statistically significant association with these phenotypes is considered a false positive. For each SNP, the FPR was defined as the proportion of replicates showing association at a significance level of 0.05. We analyzed 190 SNPs and compared the validity of HM and LRM by estimating the proportion of SNPs with FPR of greater than 5%.

In order to maintain the validity of maximum likelihood estimates (MLE) from LRM when there were no observations for a given phenotype and genotype combination, four "pseudo" subjects were added in the replicates for the type I error comparisons (190 SNPs) and power comparison only in D6. Specificially, each of these additional subjects represents 1 of the 4 possible combinations of phenotype and genotype. Both conventional LRM and HM analyses were undertaken with SAS software (version 8.2, SAS Institute, Cary, NC), and the codes for HM are available at .

## Results

**Figure 1:**
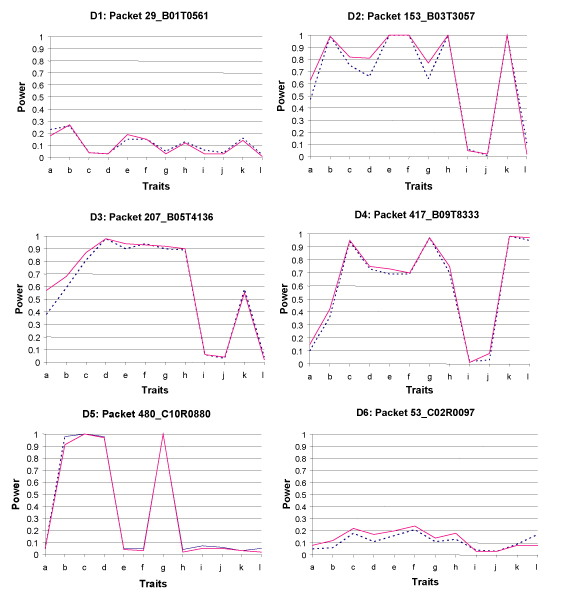
Comparison of power when evaluating multiple phenotypes: HM (solid lines) versus LRM (dotted lines).

We found that HM has slightly higher power than LRM with mean of 58.8% vs. 56.5% (Figure 1). The largest improvement was 19% for the association between phenotype *a *and D_3_. But the conventional LRM provided slightly higher power for some of the associations too, for example: phenotype *a *and D_1_. No power is shown for the random phenotypes *i *and *j*.

**Figure 2:**
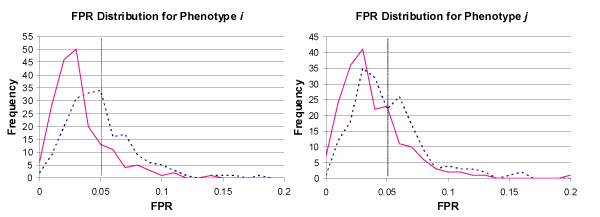
Comparison of FPR when evaluating multiple phenotypes: HM (solid lines) versus LRM (dotted lines).

More striking was the decrease in FPR provided by the HM in comparison with the LRM (to the right of the vertical line in Figure 2). With HM, 27 (14%) and 37 (19%) out of 190 SNPs total showed FPR greater than 5% for phenotypes *i *and *j*, respectively. In contrast, 61 (32%) and 70 (37%) of the tested SNPs showed FPR greater than 5% when using LRM. Testing the difference in FPR between the HM and LRM approaches for phenotypes *i *and *j *gave *p*-values < 0.001.

## Discussion

We observed that using HM to evaluate the association between SNPs and multiple disease-associated phenotypes led to substantially fewer false positives, while slightly increasing power for detecting the true associations. HM can decrease the false-positive rates because the estimate for each phenotype borrows information from other biologically similar traits, through a second-stage **Z **matrix. In the present study, we grouped phenotypes into three clusters according to the similarity of their clinical characteristics.

Note that we based our power calculations on the surrogate markers; nevertheless, the results (Figure 1) ultimately reflect the corresponding causal effect. Specifically, phenotypes *b *- *h *were controlled by D2, D3, and D4, and the markers in those regions provided power of 40–100%. In addition, only the SNP in the D4 region showed an association with the single-gene phenotype *l*, not the others. Two exceptions here are the association between phenotype *a *and the SNP in the D2 region, and an association between phenotype *k *and the SNP in the D3 region, which are not the true causal loci. This may be explained by the allelic association among these genes. For example, D1 and D3 are causally associated with phenotype *a*, and also associated with D2; therefore any association between D2 and phenotype *a *may simply reflect the effects of D1 and D3. Regardless, these relations should not affect our comparison of HM and LRM.

We did not use the "truth" for the simulations, since in practice this knowledge is not available. However, previous simulation studies based on the true underlying model between disease and exposures [4] or known LD pattern [5] indicate that hierarchical modeling offers worthwhile improvement over ordinary maximum-likelihood. Future work might also look at hierarchical modeling when the interrelated network of phenotypes and trait loci is known with certainty.

## Conclusion

Genetic association studies may attempt to reduce phenotype heterogeneity by evaluating subclinical and measurable traits instead of the primary disease of interest. Such disease-associated phenotypes may be under similar genetic control, although other genes and/or environmental factors contribute to the difference among them. By incorporating this information into a higher-level model, HM helps address problems of multiple comparisons while providing more precise estimates than conventional analyses.

## Abbreviations

FPR: False positive rates

GAW14: Genetic Analysis Workshop 14

HM: Hierarchical model

LRM: Logistic regression model

MLE: Maximum likelihood estimates

SNP: Single-nucleotide polymorphism

## Authors' contributions

XL designed the study, carried out the statistical analysis, and drafted the manuscript. EJ participated in the design of the study and helped to revise the manuscript. JSW participated in the conception, design, analysis, interpretation, and writing of the manuscript. All authors read and approved the final manuscript.
